# Supplementary material for: Resolving the full spectrum of human genome variation using Linked-Reads
Source: Genome Res. 2019 Apr;29(4):635–45. doi: 10.1101/gr.234443.118 (PMC6442396; doi:10.1101/gr.234443.118)
Supplement: Supplemental Material [file supp_gr.234443.118_Supplemental_longranger-2.2.zip › longranger-2.2.tar.bz2!/lr2.2-public/lib/go/src/code.google.com/vitess/third_party/py/mock-1.0.1.tar.gz!/mock-1.0.1/html/mocksignature.html]

mocksignature — Mock 0.8.0 documentation


### Navigation

- index
- next |
- previous |
- Mock 0.8.0 documentation »

# mocksignature¶

Note

*Autospeccing*, added in mock 0.8, is a more advanced version of
mocksignature and can be used for many of the same use cases.

A problem with using mock objects to replace real objects in your tests is that
Mock can be *too* flexible. Your code can treat the mock objects in
any way and you have to manually check that they were called correctly. If your
code calls functions or methods with the wrong number of arguments then mocks
don’t complain.

The solution to this is mocksignature, which creates functions with the
same signature as the original, but delegating to a mock. You can interrogate
the mock in the usual way to check it has been called with the *right*
arguments, but if it is called with the wrong number of arguments it will
raise a TypeError in the same way your production code would.

Another advantage is that your mocked objects are real functions, which can
be useful when your code uses
inspect or depends on
functions being function objects.

mocksignature(*func*, *mock=None*, *skipfirst=False*)¶
:   Create a new function with the same signature as func that delegates
    to mock. If skipfirst is True the first argument is skipped, useful
    for methods where self needs to be omitted from the new function.

    If you don’t pass in a mock then one will be created for you.

    Functions returned by mocksignature have many of the same attributes
    and assert methods as a mock object.

    The mock is set as the mock attribute of the returned function for easy
    access.

    mocksignature can also be used with classes. It copies the signature of
    the \_\_init\_\_ method.

    When used with callable objects (instances) it copies the signature of the
    \_\_call\_\_ method.

mocksignature will work out if it is mocking the signature of a method on
an instance or a method on a class and do the “right thing” with the self
argument in both cases.

Because of a limitation in the way that arguments are collected by functions
created by mocksignature they are *always* passed as positional arguments
(including defaults) and not keyword arguments.

## mocksignature api¶

Although the objects returned by mocksignature api are real function objects,
they have much of the same api as the Mock class. This includes the
assert methods:

```
>>> def func(a, b, c):
...     pass
...
>>> func2 = mocksignature(func)
>>> func2.called
False
>>> func2.return_value = 3
>>> func2(1, 2, 3)
3
>>> func2.called
True
>>> func2.assert_called_once_with(1, 2, 3)
>>> func2.assert_called_with(1, 2, 4)
Traceback (most recent call last):
  ...
AssertionError: Expected call: mock(1, 2, 4)
Actual call: mock(1, 2, 3)
>>> func2.call_count
1
>>> func2.side_effect = IndexError
>>> func2(4, 5, 6)
Traceback (most recent call last):
  ...
IndexError
```

The mock object that is being delegated to is available as the mock attribute
of the function created by mocksignature.

```
>>> func2.mock.mock_calls
[call(1, 2, 3), call(4, 5, 6)]
```

The methods and attributes available on functions returned by mocksignature
are:

> assert\_any\_call(), assert\_called\_once\_with(),
> assert\_called\_with(), assert\_has\_calls(),
> call\_args, call\_args\_list,
> call\_count, called,
> method\_calls, mock, mock\_calls,
> reset\_mock(), return\_value, and
> side\_effect.

## Example use¶

### Basic use¶

```
>>> def function(a, b, c=None):
...     pass
...
>>> mock = Mock()
>>> function = mocksignature(function, mock)
>>> function()
Traceback (most recent call last):
  ...
TypeError: <lambda>() takes at least 2 arguments (0 given)
>>> function.return_value = 'some value'
>>> function(1, 2, 'foo')
'some value'
>>> function.assert_called_with(1, 2, 'foo')
```

### Keyword arguments¶

Note that arguments to functions created by mocksignature are always passed
in to the underlying mock by position even when called with keywords:

```
>>> def function(a, b, c=None):
...     pass
...
>>> function = mocksignature(function)
>>> function.return_value = None
>>> function(1, 2)
>>> function.assert_called_with(1, 2, None)
```

### Mocking methods and self¶

When you use mocksignature to replace a method on a class then self
will be included in the method signature - and you will need to include
the instance when you do your asserts.

As a curious factor of the way Python (2) wraps methods fetched from a class,
we can *get* the return\_value from a function set on a class, but we can’t
set it. We have to do this through the exposed mock attribute instead:

```
>>> class SomeClass(object):
...     def method(self, a, b, c=None):
...         pass
...
>>> SomeClass.method = mocksignature(SomeClass.method)
>>> SomeClass.method.mock.return_value = None
>>> instance = SomeClass()
>>> instance.method()
Traceback (most recent call last):
  ...
TypeError: <lambda>() takes at least 4 arguments (1 given)
>>> instance.method(1, 2, 3)
>>> instance.method.assert_called_with(instance, 1, 2, 3)
```

When you use mocksignature on instance methods self isn’t included (and we
can set the return\_value etc directly):

```
>>> class SomeClass(object):
...     def method(self, a, b, c=None):
...         pass
...
>>> instance = SomeClass()
>>> instance.method = mocksignature(instance.method)
>>> instance.method.return_value = None
>>> instance.method(1, 2, 3)
>>> instance.method.assert_called_with(1, 2, 3)
```

### mocksignature with classes¶

When used with a class mocksignature copies the signature of the \_\_init\_\_
method.

```
>>> class Something(object):
...     def __init__(self, foo, bar):
...         pass
...
>>> MockSomething = mocksignature(Something)
>>> instance = MockSomething(10, 9)
>>> assert instance is MockSomething.return_value
>>> MockSomething.assert_called_with(10, 9)
>>> MockSomething()
Traceback (most recent call last):
  ...
TypeError: <lambda>() takes at least 2 arguments (0 given)
```

Because the object returned by mocksignature is a function rather than a
Mock you lose the other capabilities of Mock, like dynamic attribute
creation.

### mocksignature with callable objects¶

When used with a callable object mocksignature copies the signature of the
\_\_call\_\_ method.

```
>>> class Something(object):
...     def __call__(self, spam, eggs):
...         pass
...
>>> something = Something()
>>> mock_something = mocksignature(something)
>>> result = mock_something(10, 9)
>>> mock_something.assert_called_with(10, 9)
>>> mock_something()
Traceback (most recent call last):
  ...
TypeError: <lambda>() takes at least 2 arguments (0 given)
```

## mocksignature argument to patch¶

mocksignature is available as a keyword argument to patch() or
patch.object(). It can be used with functions / methods / classes and
callable objects.

```
>>> class SomeClass(object):
...     def method(self, a, b, c=None):
...         pass
...
>>> @patch.object(SomeClass, 'method', mocksignature=True)
... def test(mock_method):
...     instance = SomeClass()
...     mock_method.return_value = None
...     instance.method(1, 2)
...     mock_method.assert_called_with(instance, 1, 2, None)
...
>>> test()
```

### Table Of Contents

- mocksignature
  - mocksignature api
  - Example use
    - Basic use
    - Keyword arguments
    - Mocking methods and self
    - mocksignature with classes
    - mocksignature with callable objects
  - mocksignature argument to patch

#### Previous topic

Mocking Magic Methods

#### Next topic

Getting Started with Mock

### This Page

- Show Source

### Quick search


Enter search terms or a module, class or function name.

### Navigation

- index
- next |
- previous |
- Mock 0.8.0 documentation »

© Copyright 2007-2012, Michael Foord & the mock team.
Last updated on Feb 13, 2012.
Created using Sphinx 1.1.2.
